# Unique neoantigens expressed by melanomas and common epithelial cancers presented by multiple HLA alleles can be efficiently identified utilizing peptide prediction algorithms

**DOI:** 10.1186/2051-1426-3-S2-P57

**Published:** 2015-11-04

**Authors:** Kasia Trebska-Mcgowan, Mini Bharathan, Jared J Gartner, Todd D Prickett, Steven A Rosenberg, Paul F Robbins

**Affiliations:** 1 NIH/NCI, Bethesda, MD, USA; 2 Surgery Branch, National Cancer Institute, National Institutes of Health, Bethesda, MD, USA; 3 NCI/NIH, Bethesda, MD, USA

## Background

Previous observations indicate that complete regression of metastatic melanoma can be mediated by CD8 lymphocytes that predominantly recognize unique tumor specific mutations predicted in-silico to bind in context of HLA-A*02:01 [1]. The goal of this project was to expand that approach to additional alleles, as well as to evaluate it in patients with non-melanoma malignancies.

## Methods

Tumor specific mutations were initially identified by conducting whole exome sequencing and RNA sequencing on tumors and matched normal tissue. Putative minimal epitopes for each of the patients' HLA alleles were generated using in-silico MHC-peptide binding prediction algorithms (IEDB: cut off percentile rank < 2 and ann IC50 < 300nm). Candidate peptide epitopes were then pulsed onto autologous CD14+ monocytes and cultured with tumor infiltrating lymphocytes (TIL); mutation specific reactivity was detected using IFN-γ ELISA or by analysis of 4-1BB up-regulation using FACS.

## Results

Three unique neoantigens were identified as the targets of each of the 2 melanoma patients' T cell populations that were screened using this approach. Between 1 and 6 unique neoantigens, totaling 17, were identified as targets of TIL derived from 7 of the 11 patients with common epithelial cancers who were evaluated. These included 3 colorectal cancer, 2 lung cancer, 1 ovarian cancer, and 1 cholangiocarcinoma patients. No mutated target antigens were identified from three patients with colon cancer and one patient with ovarian cancer using this approach. Recognized epitopes were predicted to be presented in the context of the following class I alleles: A*01:01, A*02:01, A*03:01, A*11:01 A*30:02, B*07:02, B*40:01, B*08:01, B*57:01, C*03:03, C*06:02, C*07:01, C*07:02, C*14:02, C*16:01. Five out of the 23 mutated minimal epitopes were predicted to be the top binding candidates for the corresponding alleles, and 20 out of the 24 peptides were among the top ten of predicted binders.

## Conclusions

These observations indicate that this approach represents an efficient method for identifying cancer neoantigens restricted by multiple HLA class I alleles in patients with a variety of metastatic malignancies that include melanoma and common epithelial cancers such as lung, gastrointestinal and ovarian cancers. These studies should allow the development of novel cancer therapies based upon the adoptive transfer of enriched populations of TIL or autologous T cell receptor transduced T cells that recognize patient-specific mutated antigens.
